# Editorial: Genomics of immunoregulation and inflammatory responses in the tumor microenvironment

**DOI:** 10.3389/fgene.2023.1136342

**Published:** 2023-02-10

**Authors:** Lan Zhao, Yunpeng Cai, William C. Cho

**Affiliations:** ^1^ Department of Medicine, Stanford University School of Medicine, Stanford, CA, United States; ^2^ Shenzhen Institutes of Advanced Technology, Chinese Academy of Sciences, Shenzhen, China; ^3^ Department of Clinical Oncology, Queen Elizabeth Hospital, Hong Kong, China

**Keywords:** genomics, tumor microenvironment, integrative analysis, tumor-infiltrating cells, microbes, inflammation

The tumor microenvironment (TME) plays key roles in cancer, and targeting the TME has received significant attention in recent years. TME is characterized by infiltration of immune cells with both tumor-suppressing and tumor-promoting properties. We have edited the Research Topic (RT), entitled Genomics of immunoregulation and inflammatory responses in the tumor microenvironment with the aim to enhance our understanding of the mechanisms involving the TME as potential therapeutic targets in cancer. A total of 78 original research manuscripts and reviews were submitted between 02 November 2021 and 24 June 2022, among which 27 manuscripts were accepted for publication after peer review. Multi-omics approaches such as transcriptomics, (epi)genomics, proteomics studies have been applied for biomarker discovery and characterize the TME. The Cancer Genome Atlas (TCGA), Gene Expression Omnibus (GEO), and other public databases were used to conduct meta-analysis to identify promising mRNAs, non-coding RNAs (ncRNAs), N6-methyladenosine (m6A) RNAs, and DNA methylation-based prognostic biomarkers in patients with different types of cancer. TME components such as tumor, stromal, infiltrating immune, and vascular cells, and their positive and negative roles in cancer progression and inflammation are defined.

Gastric cancer (GC) is among the most types of cancer investigated. Both bulk and single-cell RNA-seq (scRNA-seq) methods were used to determine cancer-associated mRNA biomarkers. For example, TGFβ2 is a highly expressed gene identified from a GC bulk RNA-seq data. ScRNA-seq analysis by Wei et al. identified four high-risk genes, namely *TMPRSS15*, *VIM*, *APOA1*, and *RNASE1*, associated with poor clinical outcomes in GC. Previous study in mice has shown that *Cthrc1* expression is limited to injured vascular tissue and its involvement in arterial remodeling has been defined (LeClair et al., 2007). Combining bulk and scRNA-seq data, Zhao and colleagues found that *CTHRC1* overexpression was significantly correlated with angiogenesis, macrophage infiltration and poor prognosis in GC. Validation by immunofluorescence staining showed that *CTHRC1* was present in the vascular tissue which is considered to be involved in angiogenesis. In addition, immunohistochemistry and tissue microarray by Yu et al. indicated *INHBB* is overexpressed and correlated with poor prognosis in GC.

Besides GC, at least 10 other cancer types, and several pan-cancer studies were conducted to find new biomarkers related to prognosis, immune infiltration, and other signatures of interest. Zhang and colleagues performed an integrated bioinformatics analysis of a membrane protein, CSMD2, in pan-cancer using the TCGA, GEO, and International Cancer Genome Consortium (ICGC) databases. They found that elevated expression of *CSMD2* indicates poor prognosis and high immune infiltration levels in cancer-associated fibroblasts in multiple cancers. Another three pan-cancer studies reveal promising prognostic value and immune characteristics of CREB3L1, RUVBL2, and TLR3 respectively. Chemokines and their receptors regulate immune cell trafficking into the TME (Nagarsheth et al., 2017). CCR5 is a chemokine receptor abundantly expressed in various tumors, and has been suggested as the therapeutic targets in breast and colon cancer (Jiao et al., 2019). Through bioinformatics and experimental validations, Wang and colleagues showed that *CCR5* is upregulated at both mRNA and protein levels, and associated with poor prognosis in patients with lower-grade glioma (LGG). Five m6A and 5-methylcytosine (m5C) prognostic regulators were identified in clear cell renal cell carcinoma (ccRCC). Another integrated analysis of bulk and scRNA-seq identified HMGB3 as a hub gene associated with epithelial-mesenchymal transition (EMT) in circulating tumor cells from pancreatic ductal adenocarcinoma (PDAC).

Cancer displays heterogeneity in phenotypic properties, clinical presentations and underlying genetic causes. Molecular subtyping of cancer using data-driven and model-based methodologies offer new hope for personalized medicine (Zhao et al., 2018b; Zhao and Yan, 2019). We previously conducted an innovative molecular subtyping study in nasopharyngeal carcinoma (NPC) (Zhao et al., 2018a). We built a classifier based on a 10-microRNA (miRNA) signature to stratify NPC patients and NPC cell lines into immunogenic, classical and mesenchymal subtypes. Of note, gene set enrichment analysis (GSEA) and survival analysis indicated that the mesenchymal subtype has enriched with EMT signaling pathways and the worst clinical outcomes. A panel of 4 miRNAs were subsequently identified and employed to establish a prognostic model, by differential and uni-/multi-variate Cox regression analysis between mesenchymal and non-mesenchymal subtypes. Last, the classifier and the Cox prognostic model were assessed in both the training and validation datasets. Furthermore, heterogeneity has been observed not only in the tumor but also its microenvironment (Zhao et al., 2018c). In our current RT, there were three studies applying unsupervised hierarchical clustering of tumor-infiltrating immune cells (TIICs) to identify 2-3 immunophenotypes in lung adenocarcinoma (LUAD), bladder cancer (BLCA), and retinoblastoma, respectively.

A number of articles in the RT employed similar data processing techniques as described in our NPC study (Zhao et al., 2018a), although without proper citations. For example, Zou and colleagues selected 136 apoptosis-related genes in the KEGG apoptosis pathway (map04210), and identified 64-gene that were differentially expressed between tumor and non-tumor tissues in LUAD from the TCGA database. Unsupervised consensus clustering was applied to classify the patients into two prognostic subtypes based on these 64-gene signatures. Differential and Cox regression analysis between the two subtypes helped to identify and build a 11 apoptosis-gene signature model for survival risk prediction. The model was further validated in the patient and cancer cell line datasets. Another study in LUAD identified three 7-methylguanosine (m7G)-associated prognostic signatures.

Similar approaches were conducted in gastric and colorectal cancers. The authors focused on necroptosis- and panoptosis-related genes, respectively, and their contributions to the TME in their respective cancers. The two studies also performed comprehensive bioinformatics and quantitative real-time polymerase chain reaction (qRT-PCR) experimental validations, which may represent improvements in this field but still lack some novelties. Another set of unique necroptosis-related gene signatures were identified in diffuse large B cell lymphoma although without significant advances in terms of data processing and analysis.

Ferroptosis- and pyroptosis-related prognostic gene signatures were identified from another four (Han et al., Huang et al., Wang et al., and Liu et al.) and Zhong et al.’s studies, respectively, using similar or identical data processing steps. The gene signatures in these four studies (Han et al., Huang et al., Wang et al., and Zhong et al.) were long non-coding RNAs (lncRNAs), and no overlaps were found between any of the studies. RNAs longer than 200 nucleotides in length that do not encode proteins are broadly categorized as lncRNAs. In contrast to mRNAs, lncRNAs are less abundant, less evolutionarily conserved, more tissue-specific, and are localized predominantly in the nucleus (Statello et al., 2021). The discovery of lncRNAs and their diverse functions in various cellular processes, including oncogenic signaling, have provided new perspectives in cancer prognosis (Evans et al., 2016; Bridges et al., 2021). In the current RT, the above four studies noted the important immune-related roles of lncRNAs in the TME, and there is another study in breast cancer which emphasized the contribution of lncRNAs in amino acids metabolism. Altered metabolism is a hallmark of cancer. The role of amino acids and lipids in cancer metabolism is very much appreciated recently (Lieu et al., 2020; Snaebjornsson et al., 2020). A total of two studies in this RT identified metabolism-related gene signatures for predicting the prognosis of patients with breast and head and neck cancer, respectively. Although interesting, the two pure bioinformatics studies lacked experimental validations.

Considering there are at least 9 papers in the RT mentioning apoptosis, necrosis, ferroptosis, and other forms of cell death, it is necessary to add a paragraph or two to talk about the subject in terms of comparing their characteristics and roles in cancer. Apoptosis is a highly regulated and conserved form of normal programmed cell death (PCD) in multicellular organisms (Kerr et al., 1972). The apoptotic pathway is mainly activated by the intrinsic mitochondrial and extrinsic death receptor signals (Wong, 2011). In addition, under endoplasmic reticulum (ER) stress, the unfolded protein response (UPR) pathway is central for apoptotic cell death (Hetz et al., 2020). In this RT, there is a study carried out by Zhang and colleagues to identify UPR-related prognostic signatures in Osteosarcoma. Necrosis is an inflammatory-inducing cell death, which has been characterized as an accidental and passive process that results from external causes of injury (Syntichaki and Tavernarakis, 2002). The dysregulation of apoptosis and the harsh necrotic microenvironment contribute to malignant transformation and progression. For example, it is well-known that apoptosis evasion is a hallmark of cancer. Cancer cells evade apoptosis either by overexpressing anti-apoptotic genes such as *BCL2* or silencing the expression of pro-apoptotic transcription factor *TP53* (Fernald and Kurokawa, 2013). Hypoxia and metabolic stress induce necrosis and provoke immune responses, which eventually lead to cancer formation and TME modification (Karsch-Bluman et al., 2019). Moreover, a necroptosis-associated cytokine microenvironment regulates liver cancer progression (Seehawer et al., 2018). Ferroptosis is an non-apoptotic, iron-dependent, and oxidative form of cell death (Dixon et al., 2012). Iron has been found to play a role in the TME and in metastasis (Torti and Torti, 2013). Thus, iron and ferroptosis are promising therapeutic targets in cancer. In our current RT, Huang and colleagues identified and validated a 3-ferroptosis-related lncRNA prognostic signature in glioma. They performed functional and biochemical analysis of one of the signatures, LINC01426, which can be useful for future references.

A number of pathogens such as enteric *Salmonella* and *Shigella* species can induce a novel form of PCD, namely pyroptosis, in infected host cells (Boise and Collins, 2001; Fink and Cookson, 2005; Jorgensen and Miao, 2015). Although pyroptosis is inflammatory, it differs from necrosis which is caspase dependent and genetically controlled (Boise and Collins, 2001; Fink and Cookson, 2005). Detailed and comprehensive discussion of apoptosis, necrosis, ferroptosis, pyroptosis and their roles in cancer can be found in reviews (Boise and Collins, 2001; Fink and Cookson, 2005; Yu et al., 2017). The four forms of cell death may act as defense systems against microbial infection (Fink and Cookson, 2005; Amaral et al., 2019). Crosstalk between microbes and hosts, in terms of microbe-induced host cell death and cancer prevention and survival outcomes, can be further expanded in future studies.

Taken together, the TME is characterized by infiltrated immune cells that can influence cancer progression and patient prognosis. The connection between chronic inflammation in TME and cancer is bidirectional and complex. Large amounts of genomics data deposited in public repositories advance the fields of oncology and immunology. Uncovering novel risk-associated biomarkers in cancer through systematic review and meta-analysis are necessary and proven to be useful.
